# DNA methylation analysis of the epigenome in oral squamous cell carcinoma

**DOI:** 10.1186/s40246-025-00899-3

**Published:** 2026-01-05

**Authors:** Jun Ma, Yu Huang, Yuxin Bai, Na Zhou, Hanxuan Wang, Ying Zhang, Min Hu, Jiaqing Yan

**Affiliations:** 1 Hospital of Stomatology, Jilin University, Changchun, 130021 People’s Republic of China; 2 Jilin Provincial Key Laboratory of Tooth Development and Bone Remodeling, Hospital of Stomatology, Jilin University, Changchun, 130021 People’s Republic of China; 3 Faculty of Chinese Medicine, Macau University of Science and Technology, Macau, 999078 China; 4 School of Pharmacy and Laboratory of Drug Discovery from Natural Resources and Industrialization, Macau University of Science and Technology, Macau, 999078 China; 5 State Key Laboratory of Mechanism and Quality of Chinese Medicine, Macau, 999078 China

## Abstract

**Background:**

Oral squamous cell carcinoma (OSCC) is one of the most common oral malignancies, which can occur in any part of the mouth and is highly malignant. DNA Methylation is an epigenetic modification of the genome, which is involved in key cellular processes and has a crucial impact on the occurrence, development, invasion and metastasis of tumors. In this study, we conducted a comprehensive analysis of DNA methylation characteristics in OSCC with the aim of identifying potential diagnostic epigenetic biomarkers and exploring possible mechanisms of methylation’s influence on OSCC.

**Methods:**

In this study, genome-wide DNA methylation analysis was performed using Infinium Methylation EPIC arrays, including tumor tissue and adjacent non-tumor tissue from 12 OSCC patients. Differential methylation probes and regions (DMP/DMR) were identified for gene function analysis. Characteristic DMPs and genes were screened according to the specific situation, and OSCC-targeted methylation data from 25 patients in the validation cohort were used to further validate the differential methylation levels of our selected genes. Finally, the expression levels of methylated genes in OSCC were verified by combining RNA-Seq data with quantitative real-time polymerase chain reaction (qRT-PCR).

**Results:**

There were 277,805 DMPs in OSCC tumor tissue. Hypermethylated DMP accounted for 37.4% of all DMPs and hypomethylated DMPs was 62.6%. Functional pathway analysis showed that it was mainly related to passive transmembrane transporter activity, cancer proteoglycan and PI3K-Akt signaling pathway. The methylation level of ZNF880 was emphatically verified in the verification cohort, and the results showed that there was high methylation in ZNF880 in the verification cohort. Subsequently, through RNA-Seq data and qRT-PCR, it was confirmed that the expression of ZNF880 in OSCC tissues was significantly lower than that in normal tissues. This verified the correlation between the high methylation of ZNF880 and gene expression.

**Conclusions:**

This study comprehensively reveals changes in genome-wide DNA methylation patterns in OSCC, indicating that abnormal hypermethylation of the ZNF880 gene plays a catalytic role in the pathogenesis of OSCC.

**Supplementary Information:**

The online version contains supplementary material available at 10.1186/s40246-025-00899-3.

## Introduction

Oral squamous cell carcinoma (OSCC), the most prevalent oral malignancy accounting for over 90% of oral cancers, ranks as the sixth most common cancer globally [1]. In 2022, an estimated 389,485 new patients were diagnosed with lip and oral cancer worldwide, with an estimated 188,230 deaths [2]. OSCC can occur anywhere in the mouth, including the tongue, upper and lower gums, floor of the mouth, palate, and buccal mucosa [3]. This type of cancer is more aggressive, and the early onset is more insidious. Most patients are found in the middle and late stages, which leads to a reduced survival rate [4], and the five-year survival rate is often less than 50% [5]. The metastases of OSCC patients are associated with prognosis. Therefore, early diagnosis is extremely important for the follow-up treatment of tumors and the prognosis of patients [6]. It can be seen that early diagnosis of OSCC has become an urgent goal.

Many studies have shown that DNA methylation is an epigenetic modification of the genome, which is involved in key cellular processes, such as transcription, embryonic development, and normal regulation of chromatin structure [7]. In tumor tissues, DNA methylation also plays an important role in the occurrence, development, invasion, metastasis and other processes of tumors [8]. Looking for specific markers of abnormal methylation at an early stage has become a viable way to diagnose OSCC, as molecular changes are often more sensitive than cell morphological changes, and changes that affect the course of the disease have already occurred when there are precancerous lesions or no cancer cells [9].

In summary, this study was conducted to investigate DNA methylation profiles and their changes in OSCC and adjacent samples, and to identify differential methylation probes (DMPs) specifically associated with OSCC. The results of this study contribute to a better understanding of the relationship between DNA methylation and OSCC, and also help identify potential diagnostic markers and potential therapeutic targets for OSCC.

## Materials and methods

### Subjects

This study included 37 patients diagnosed with OSCC in the oral and maxillofacial surgery of Hospital of Stomatology in Jilin University in China during 2020 and 2022 years. Inclusion criteria: (1) Patients had clinical symptoms and signs of OSCC; (2) OSCC was confirmed by pathological biopsy, and no other diseases were associated; (3) All patients were newly diagnosed with OSCC and did not receive radiotherapy or chemotherapy before surgery. This study was approved by the Ethics Committee of Hospital of Stomatology Jilin University (No.:201963) and was conducted in accordance with the principles outlined in the *Declaration of Helsinki*. Informed consent was obtained from all participants involved in the study. In each patient, the diseased tissue and adjacent normal tissue were taken simultaneously by surgery. The samples were dried with sterile gauze and cut into tissue blocks with a thickness of less than 0.5 cm. After the blood stains and non-essential tissues were washed away, the samples were placed in labeled DNase/ RNase-free freezing tubes. After the liquid nitrogen was quick-frozen, the tubes were stored in -80 °C for further treatment.

### DNA isolation, extraction and bisulfite modification

DNA isolation was performed on the QIAamp Fast DNA Tissue Kit (Qiagen, Germany) according to the manufacturer’s instructions. In short, 5 mg OSCC and ANT samples were ground with nitrogen and then placed in a tissue crushing tube. Next, 300 µL of the main mixture containing the cleavage buffer, protease K, and RNase A were added. The tissues were then homogenized in a bead mill at 45 Hz for 2 min and the tubes were incubated in a hot mixer at 1000 rpm and 56 °C for 30 min. After the DNA was collected and washed with the QIAamp Mini centrifugal column, the genomic DNA was finally collected into a 1.5 ml EP tube. A NanoDrop ND-1000 spectrophotometer (NanoDrop Technologies Inc., USA) was used to measure DNA concentration and purity in a liquid.

### DNA methylation analysis

Genome-wide DNA methylation patterns of tissue samples were obtained using the Illumina Methylation EPIC (850 K) BeadChip platform, which enables DNA methylation analysis of more than 850,000 CpG sites. Bisulfite treatment, genome-wide DNA amplification, hybridization and single base extension, fluorescent staining, and chip scanning were all performed according to manufacturer (Illumina)’s instructions. In short, 250 ng of genomic DNA extracted from frozen tissue samples was converted by bisulfite using the EZ-96 DNA methylation kit (deep hole, Zymo Research Corporation). The samples were hybridized on the Infinium Methylation EPIC BeadChip. DNA methylation was measured according to the manufacturer’s protocol using Infinium Methylation EPIC BeadChip.

In order to obtain high-quality data, the ChAMP package in R software (version 4.4.0) was used for quality control (QC) and pre-processing of the original data. Samples that did not meet the following quality control standards were excluded: (1) probes that detected P-values > 0.01 in one or more samples; (2) probes with beads < 3 in at least 5% of samples; (3) non-CpG probes; (4) probes with single nucleotide polymorphisms (SNPs); (5) probes aligned to multiple locations; (6) sex chromosome specific probes. All these probes were removed from subsequent analyses. The β value represented the percentage of methylation at each CpG site on the Methylation EPIC BeadChip, and these values ranged from 0 (completely unmethylated) to 1 (fully methylated). The β-value matrix was then normalized using the Beta-Mixture Quantile (BMIQ) method, a model-based normalization method used to correct the β-value of the Type II probe based on the beta distribution of the β-value of the Type I probe. In addition, the batch effects caused by the BeadChip Slide and array were corrected using Combat, and the adjusted P-values were calculated using the Benjamini-Hochberg method.

### Differential methylation probes and regions (DMPs/DMRs) screening

Inter-group differential methylation was defined as |beta difference | >0 and adjusted p value (Benjamini-Hochberg method) < 0.05. Similarly, the methylation region with P value < 0.05 after final correction is considered to be the differential methylation region [10].

### Annotation and functional analysis of DMPs and DMRs

Next, the resulting DMPs and DMRs were commented. In short, GenomicRanges package in R were used to identify differentially methylated genes and determine the nearest transcription start site (TSS) and genetic composition, and then enrichment analysis was performed for DMPs and DMRs related genes. Gene Ontology ( GO ) biological processes and Kyoto Encyclopedia of Genes and Genomes (KEGG) pathway analysis were performed using the ClusterProfiler package in R.

### Screening and verification of characteristic DMPs and their corresponding genes

In order to find the characteristic DMPs strongly associated with OSCC and their corresponding genes, DMPs meeting the following two points were considered for subsequent analysis: (1) The first 100 DMPs were taken after all the DMPs were arranged according to the difference of β values in descending order [11], (2) the DMPs in the non-TSS were excluded, (3) the highly methylated DMPs in normal tissue were excluded, and (4) the most significant 5mC sites span genomic regions approximately 500 bp in length. For the obtained feature DMPs, Massarray analysis was performed on all CpG islands in the DNA of their corresponding genes in the validation group to verify the degree of methylation. In short, the sulfite treated DNA was extended and quantitative analysis of DNA methylation was performed using the MassARRAY EpiTYPER platform (Sequenom, San Diego, CA, USA) [12–14]. Primer sequences for sulfite PCR are available in supplementary Table 1.

### Collection and processing of data from public databases

The methylation data and clinical data of OSCC patients from the TCGA-HNSC cohort were retrieved through the cancer genomics data portal ( https://portal.gdc.cancer.gov/ ). The processing standards for the methylation data remain the same as before. The transcriptome data of datasets GSE160042, GSE23558, and GSE30784 were retrieved from the GEO database ( https://www.ncbi.nlm.nih.gov/geo/ ) and merged. Given the potential batch effects across different datasets, the “ComBat” algorithm in the “sva” package was employed to eliminate them.

### Survival analysis and clinical correlation analysis of methylation

Clinical pathological features and survival data were extracted from TCGA to explore the relationship between the methylation level of characteristic genes and different clinical pathological features. Patients with OSCC were divided into high methylation group and low methylation group according to the optimal cut-off value of methylation level in tumor tissues. Kaplan-Meier curves were plotted using the “survival”, “survminer” and “forestplot” packages, and differences were compared by the log-rank test. Meanwhile, univariate and multivariate COX regression analyses were employed to investigate whether the methylation level serves as an independent prognostic factor. Then, receiver operating characteristic (ROC) curve analysis was used to evaluate the discriminative ability and accuracy of the methylation level of the characteristic gene.

### Differential expression of characteristic genes between OSCC tissues and normal tissues

Transcriptome data from GEO datasets were used to compare the differential expression of characteristic genes between OSCC tissues and normal oral tissues, and boxplots were drawn using the “ggplot2” package.

### Real-time quantitative PCR

RNA was extracted from normal tissues and OSCC tissues of 6 patients using the SteadyPure RNA Extraction Kit (AG21101-C; Accurate Biology, China). cDNA was synthesized using the Evo M-MLV Reverse Transcription Premix Kit (AG11718; Accurate Biology, China). Quantitative RT-PCR was performed using the SYBR Green Pro Taq HS PreMix qPCR Kit (AG11701; Accurate Biology, China). The primer sequences were consistent with those used previously.

### Statistical analysis

The GraphPad Prism software (www.graphpad.com, version 7) and RStudio (www.rstudio.com, version 4.4.0) in the R statistical environment were utilized to perform statistical analysis and data visualization tasks. Differential analysis of methylation data was performed with ChAMP (version 2.34.0). GenomicRanges (version 1.58.0) were used to determine the nearest TSS and genetic composition. GO biological processes and KEGG pathway analysis were performed with ClusterProfiler (version 4.14.3). Batch effects were eliminated using the sva package (version 3.54). ROC curves were plotted using the pROC package (version 1.18). Kaplan-Meier curves were drawn using the “survival” package (version 3.5), “survminer” package (version 0.5.6) and “forestplot” package (version 1.5.2). Boxplots were plotted using the “ggplot2” package (version 3.5.2). Statistical analysis was performed using bilaterally paired student t test. A statistical significance threshold of *p* < 0.05 was established.

## Results

### Patient characteristics

According to the above inclusion criteria, a total of 37 patients with OSCC were enrolled in this study, which was divided into two phases. In the first stage, 12 patients were used as an experimental group to measure the methylation of tumor tissue and adjacent tissue. In the second phase, the remaining 25 patients were used as validation groups to verify the degree of methylation of the genes. The demographic characteristics and pathological types of all patients are found in supplementary Table 2.

For the TCGA database, methylation data of OSCC in TCGA-HNSC were obtained, including 361 OSCC tumor tissues and 34 normal tissues. For the GEO database, transcriptome data from three datasets were merged, and the merged dataset included 204 tumor tissues and 59 normal tissues.

### Quality control results of samples and probes

**Fig. 1:**
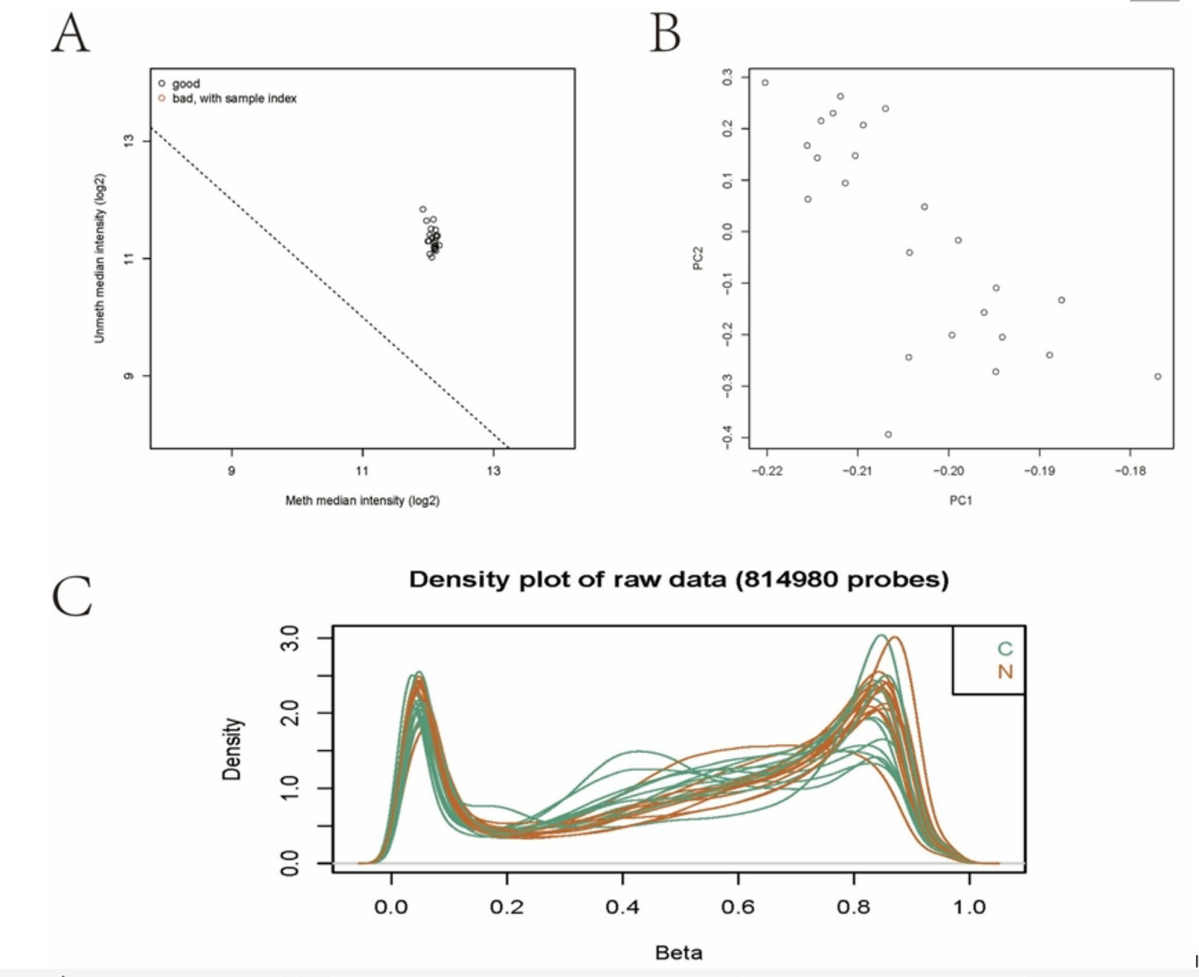
**A** Quality control of the overall sample signal.** B** Principal Component Analysis (PCA) plot.** C** Probe β-value density plot

After standardizing the raw data obtained, all samples were evaluated for quality. The results showed that all samples were of good quality and in line with quality control standards (Fig. 1A). PCA analysis showed no significant batch effect between samples (Fig. 1B). The β-value density curve showed that the methylation levels of tumor tissue samples and normal samples were bimodal and did not differ greatly among the samples (Fig. 1C). Therefore, in general, the quality of the sample is acceptable and can be analyzed in the next step.

### Differential methylation probes (DMPs) and region (DMRs) analysis

**Fig. 2:**
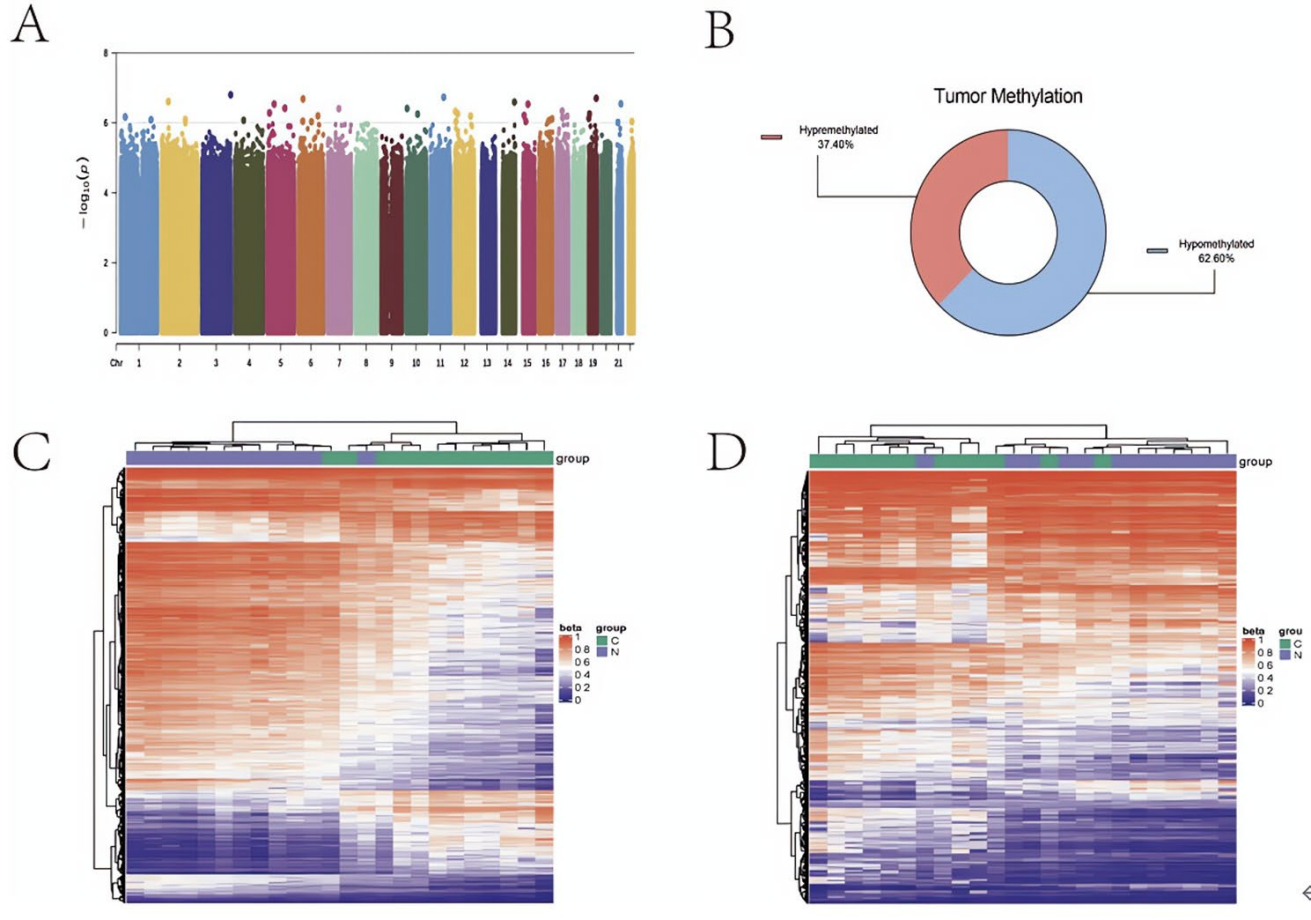
**A** Overall methylation status.** B** Distribution of methylation levels.** C** Differential methylation probes (DMP) clustering heatmap situation.** D** Differential methylation regions (DMR) clustering heatmap situation

A total of 814,980 CpGs were detected in 12 tumors and normal samples. According to the DMPs screening criteria mentioned above, all the methylation sites were filtered, and 277,805 DMPs were obtained. These DMPs were widely present on all chromosomes (Fig. 2A). Notably, the DMPs for hypermethylation in OSCC tumor tissues (104,022, 37.4% of all DMPs) was lower than that for hypomethylation (62.6%) in normal tissues (Fig. 2B). Complete DMPs information is available in supplementary Table 3. After further screening of DMRs according to the above criteria, 686 significant DMR were obtained, including 440 hypermethylated DMRs and 146 hypomethylated DMRs. Complete DMRs information is stored in supplementary Table 4. Next, the overlapping parts of DMPs and DMRs were clustered, and the unsupervised hierarchical clustering showed obvious clustering between normal and tumor samples, indicating that methylation can distinguish OSCC well (Fig. 2C, D).

### GO and KEGG analysis of DMRs

**Fig. 3:**
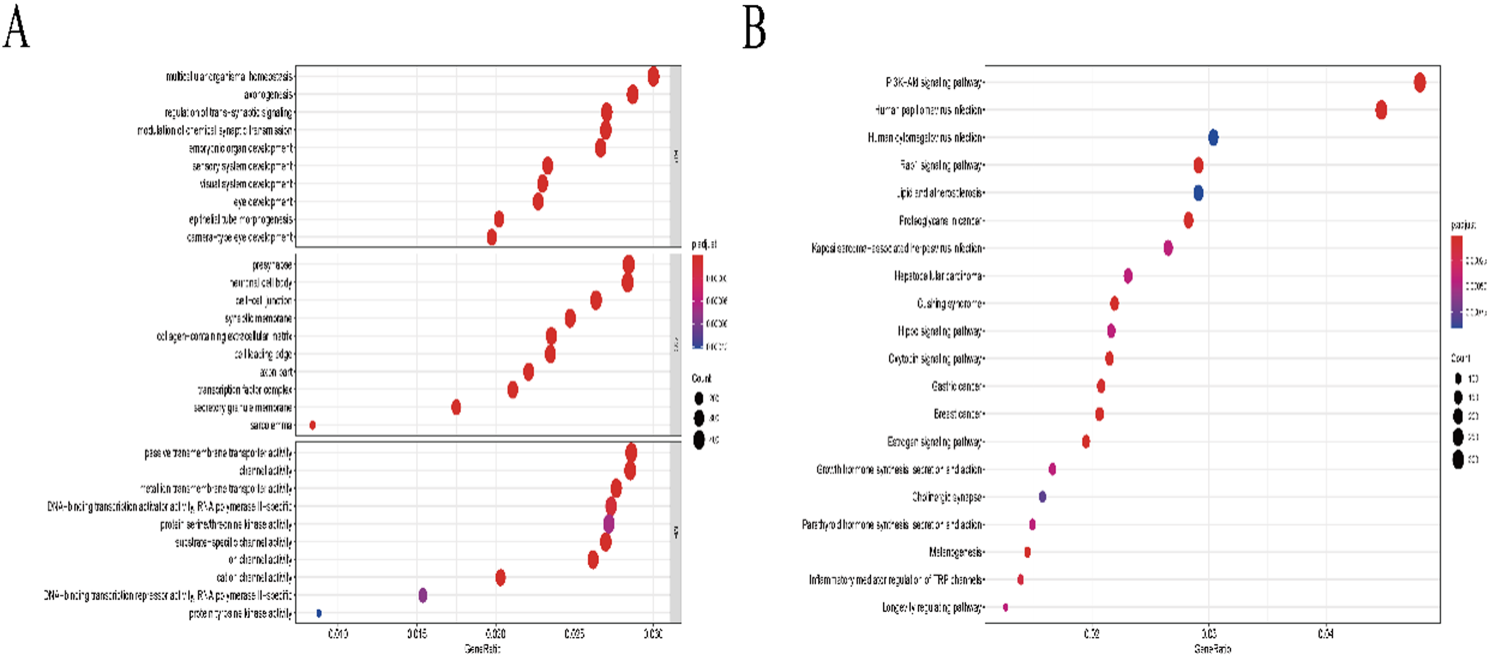
**A** GO enrichment analysis.** B** KEGG enrichment analysis. The Y-axis represents the enriched GO terms and KEGG pathways, while the X-axis represents GeneRatio, which is the percentage of genes associated with each GO or KEGG term. The size of the dots indicates the Count (the number of genes associated with each term)

To further investigate the potential impact of genome-wide DNA methylation changes in OSCC, we performed GO bioprocess and KEGG pathway enrichment analyses for DMPs and DMRs. The results of GO analysis included three functional groups — biological processes, molecular functions, and cellular components. In terms of biological processes (BP), these genes are mainly involved in the regulation of homeostasis and synaptic transmission in multicellular organisms. Cell component (CC) enrichment analysis showed that presynaptic and neuronal cell bodies were associated with these genes. Molecular function (MF) analysis showed that these genes were mainly enriched in passive transmembrane transporter activity and channel activity (Fig. 3A). In addition, in the category of KEGG pathway analysis, it was mainly related to the PI3K-Akt signaling pathway, cancer proteoglycans, inflammatory mediators’ regulation, and Rap1 signaling pathway (Fig. 3B).

### Verification of characteristic DMPs and its corresponding genes

**Fig. 4:**
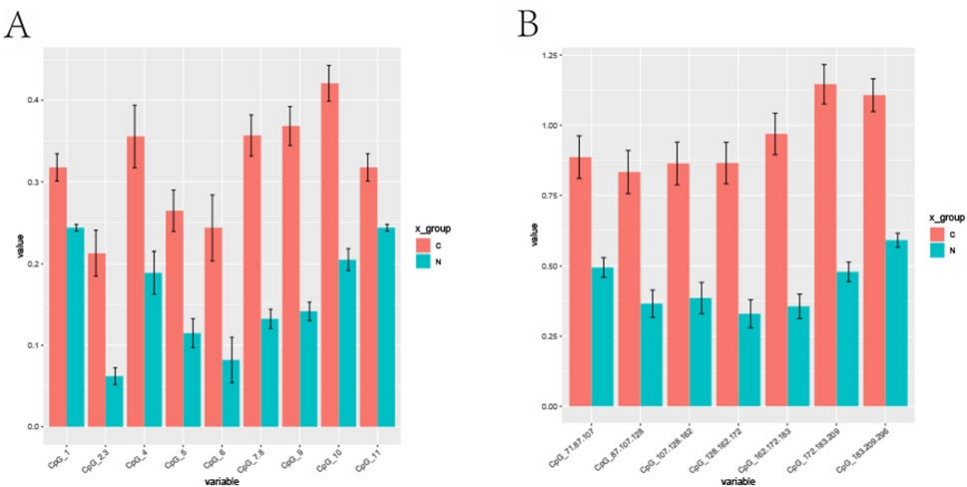
**A** Average level of methylation at methylated sites** B** Average level of methylation in methylated regions. The methylation level of ZNF880 in tumor tissues is higher than that in normal tissues

Based on the above screening criteria, the obtained DMPs were screened, and finally, three feature DMPs highly related to methylation were obtained: cg02948555 (corresponding to the ZNF106), cg01572741 (corresponding to the ZNF880), and cg11275536 (corresponding to the ASAP1). In the analysis of the discovery cohort, the cg01572741 site corresponding to ZNF880 exhibited a more pronounced methylation difference, with an average methylation difference value of 0.474, higher than that of ZNF106 (0.433) and ASAP1 (0.289), and demonstrated stronger statistical significance (*p* = 1.75E-06, supplementary Table 3). Additionally, this site is located in the TSS1500 region near the transcription start site and the Shore region of a CpG island. The association between methylation changes in Shore regions and tumor development has been substantiated by multiple studies [15, 16], suggesting that this site may have greater functional regulatory significance. Therefore, in the subsequent validation phase, we prioritized ZNF880 and assessed its methylation level in an independent validation cohort using MassArray technology. The results showed that the methylation level of ZNF880 in tumor tissues was significantly higher than in normal tissues in the validation cohort (Fig. 4A, B).

### Lack of correlation between ZNF880 methylation level and clinical status

Due to the small sample size, we further used TCGA-OSCC methylation data to verify the methylation level of ZNF880 in a larger cohort (tumor = 361, normal = 34). The results showed that the methylation level of ZNF880 in tumor tissues was still significantly higher than that in normal tissues (Supplementary Fig. 1). This finding was consistent with our results. Next, we analyzed the correlation between ZNF880 methylation levels and patients’ clinical characteristics. The results showed that there was no significant difference in ZNF880 methylation levels among patients with stage I–IV disease (Supplementary Fig. 2A). Similarly, no significant differences were observed in terms of TNM staging (Supplementary Fig. 2B–D).

### Diagnostic and prognostic significance of ZNF880 methylation level in patients

**Fig. 5:**
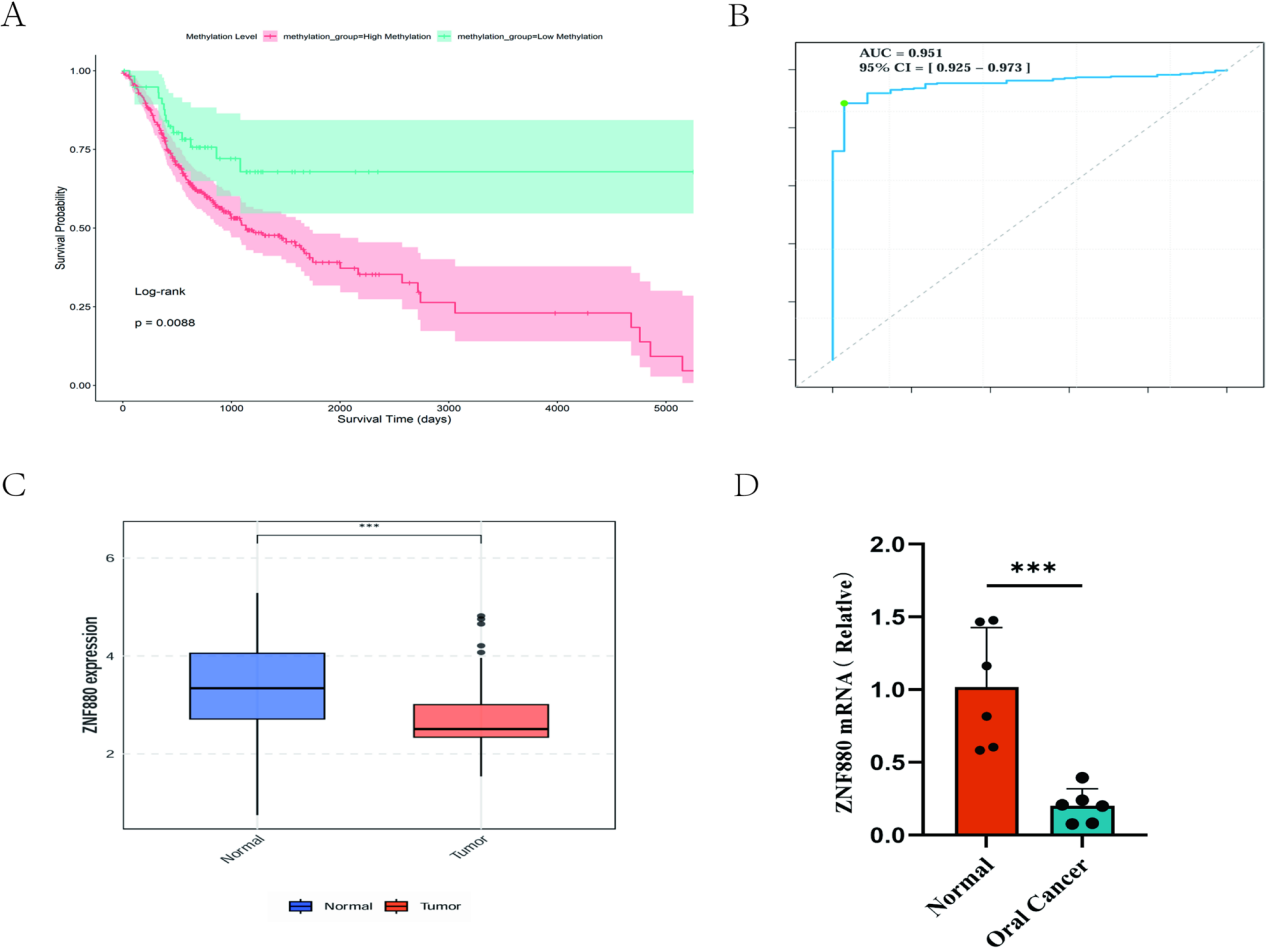
**A** Kaplan–Meier analysis of ZNF880 methylation in the OSCC cohort from TCGA.** B** ROC curve of ZNF880 methylation.** C** Significantly downregulated ZNF880 expression in tumor tissues compared with normal tissues of OSCC patients in the GEO database.** D** Total ZNF880 levels in tumors (*n* = 6) were significantly downregulated compared with adjacent normal tissues (*n* = 6). ns = not statistically significant, = *p* < 0.05, = *p* < 0.01, = *p* < 0.001, ****= *p* < 0.0001. Data are presented as mean ± SEM

We extracted clinical data from the TCGA-OSCC cohort, excluding patients with a survival time of less than 30 days. Patients were stratified into high and low methylation groups based on the optimal cut-off value determined for Kaplan-Meier survival analysis. The results revealed a significant correlation between ZNF880 methylation levels and overall survival (OS), with the low methylation group exhibiting significantly better OS compared to the high methylation group (Fig. 5A). Further analysis of disease-specific survival (DSS) and progression-free survival (PFS) in relation to methylation levels demonstrated a significant association between ZNF880 methylation and PFS, with the low methylation group showing markedly better PFS than the high methylation group (Supplementary Fig. 3A). Although no statistically significant difference was observed for DSS, a similar trend to that of OS and PFS was noted (Supplementary Fig. 3B). Subsequent univariate and multivariate Cox regression analyses of ZNF880 methylation levels and OS identified high ZNF880 methylation as a significant risk factor for OS (Supplementary Fig. 4A, B). Additionally, receiver operating characteristic (ROC) curve analysis was conducted, yielding an area under the curve of 0.951 (95% CI: 0.925–0.973), indicating that ZNF880 methylation has robust diagnostic performance (Fig. 5B).

### Verification of ZNF880 gene expression

To further verify whether the high methylation of the ZNF880 gene silences its expression at the gene level, we conducted differential analysis using RNA-seq data from 204 tumor tissues and 59 normal tissues in the dataset. The results showed that the expression of ZNF880 in tumor tissues was significantly lower than that in normal tissues (Fig. 5C). Subsequent qRT-PCR results showed the same trend (Fig. 5D). These results collectively indicate that increased ZNF880 methylation indeed silences the expression of the ZNF880 gene.

## Discussion

Since OSCC is a highly recurrent malignancy, its early diagnosis is particularly important. With the advent of a new generation of DNA methylation chips, Illumina Infinium Methylation EPIC BeadChip, more CpG probes can be measured. On this basis, this study used EPIC array to conduct genome-wide methylation studies on OSCC, covering more than 850,000 CpG probes, including CpG islands, gene promoter region, enhancer region and gene coding region. In this study, it was found that in OSCC tumor tissues, hypermethylated DMPs accounted for 37.4% of all DMPs, lower than 62.6% of hypomethylated DMPs, which was similar to previous studies [10]. It is worth noting that the patients included in this study are Han people in China, and further verification has been conducted in the European white population. Notably, this substantially reinforces the reliability of our findings across diverse ethnic populations. To our knowledge, the present study represents the first genome-wide DNA methylation investigation of oral squamous cell carcinoma involving individuals of Han Chinese ancestry.

In recent decades, genomic and epigenomic characterization has led to new understanding of the carcinogenic role of certain cancers and facilitated diagnosis and personalized treatment strategies. DNA methylation is the most widely studied epigenetic mechanism and is known to play a key role in inhibiting gene expression as well as maintaining the stability of their genomes. It is generally believed that the high methylation of tumor suppressor genes in OSCC may be related to gene silencing and early tumor formation [17]. In recent years, there have been many studies on OSCC methylation, and researchers tend to find some reliable methylation-related genes as biomarkers for the diagnosis or treatment of OSCC [18]. Previous studies of OSCC have mainly aimed at identifying changes at a small number of sites in the genome or using low-throughput methylation arrays [19–21]. With the development of methylation technology, high-throughput methylation sequencing technology has been gradually used. Krishnan et al. [22] used HumanMethylation450K BeadChip (Illumina) to analyze genome-wide methylation changes in oral tongue squamous cell carcinoma (OTSCC), but only about half of the sites were considered compared to this study due to the limitations of chips and probes. Gonzalez et al. [10] further used HM850K to analyze genome-wide methylation changes in OTSCC, but the sample size seemed to be small (*N* = 6), and primary tumors in other parts of the mouth were not considered. Building on previous studies, this study was further refined to include more samples and take into account squamous cell tumors anywhere in the mouth.

The results of this study showed 277,805 DMPs and 686 significant DMRs, including 440 hypermethylated DMRs and 146 hypomethylated DMRs. The enrichment results showed that methylation-related genes were mainly related to the regulation of homeostasis and synaptic transmission, the activity of passive transmembrane transporter and channel in presynaptic and neuronal cell bodies. In terms of pathway, it is related to PI3K-Akt signaling pathway, cancer proteoglycans, inflammatory mediators regulation, and Rap1 signaling pathway, which is also similar to previous experimental studies [23–25]. This indicates that the methylation process is related to the occurrence, development, invasion and metastasis of OSCC.

In order to identify specific biomarkers for the diagnosis of GC, strict criteria were developed in this study. First, the DMPs with the largest difference was selected after the difference values (β) being arranged from the largest to the smallest. Then, the most significant probes of 5mC were selected as the main sites, which could cover the fragment length of about 500 bp. Finally, three genes with diagnostic efficacy were identified: ZNF106 is an RNA-binding protein that binds to core splicing factor RNA-binding motif protein 39 and is localized to a nuclear spot near the spliceosome [26]. Recent studies have shown that ZNF106 has good prognostic value in esophageal and bladder cancer [27, 28]. However, it is worth noting that there is currently no research investigating the role of ZNF106 in OSCC. ZNF880 is a member of the KZNFs family, and some studies suggest that KZNFs genes act more as tumor suppressor genes [29, 30]. ASAP1 is a member of the ARF-GAPS family and participates in membrane-cytoskeletal interactions that affect membrane motion, cell appearance, and motion [31].In a recent study, Sato et al. [32]. found that elevated expression of AMAP1 (also known as ASAP1) in OSCC patients may be associated with poorer prognosis. They proposed a hypothesis that ASAP1, as a downstream effector, may participate in the Arf6 pathway and play a crucial role in the invasion and metastasis of OSCC cells.

Notably, based on the criteria selected in this study, three genes with a high correlation with methylation levels were finally identified and the methylation levels of ZNF880 were validated in the validation cohort. ZNF880 remained hypermethylated in the validation cohort, which guaranteed consistency of results. Subsequently, in the broader TCGA-OSCC cohort, the methylation level of ZNF880 in tumor tissues remained significantly higher than that in normal tissues. Moreover, the prognosis of the high methylation group was significantly poorer. These results suggest that ZNF880 may exert important prognostic value in OSCC, which could provide a reference for the long-term treatment of OSCC patients. Interestingly, the present study indicates that ZNF880 methylation possesses favorable diagnostic performance, which may offer new insights into the early diagnosis of OSCC.

ZNF880 has been poorly studied in OSCC. Zinc finger proteins (ZNFs) constitute the largest family of sequence-specific DNA-binding proteins. They bind to target DNA sequences via zinc finger domains and are involved in numerous life functions, such as signal transduction and prevention of DNA breakage [33]. In previous studies, ZNF880 was found to be significantly down regulated after hypermethylation in colorectal cancer and was significantly associated with overall survival (OS) and disease-free survival (DFS) [34]. This is similar to our research results. This indicates that high methylation of ZNF880 is associated with poor tumor prognosis. Notably, in the same study, Dong et al. demonstrated that ZNF880 methylation can regulate the upregulation of CDK1, a key cell cycle regulatory protein that plays a crucial role in different stages of the cell cycle. It is closely associated with cell cycle transitions, including the G1/S and G2/M transitions [35]. A large number of studies have demonstrated that CDK1 also plays a significant role in OSCC, including promoting the growth, invasion, and metastasis of tumors [36, 37]. Thus, we hypothesize that in OSCC, ZNF880 methylation may also promote tumor biological processes by regulating CDK1. However, there is currently no direct evidence linking ZNF880 methylation levels to CDK1 expression, and the specific molecular mechanism by which ZNF880 methylation regulates CDK1 remains unclear. This may be a direction for our future research. Abnormal hypermethylation tends to silence gene expression. In another study, ZNF880 showed significant methylation changes and significantly down-regulated expression in head and neck squamous cell carcinoma [38]. In this study, we validated that the expression of ZNF880 in tumor tissues is lower than that in normal tissues using both the GEO database and clinical samples. This further strengthens the association between methylation levels and gene expression. Currently, some epigenetic drugs have been approved by the FDA for the treatment of tumors [39]. Members of the ZNF family (such as ZNF382) have been proven to be able to restore normal methylation through epigenetic drugs, thereby inhibiting the tumor growth process [40]. Unfortunately, however, there appears to be no similar research on ZNF880 so far. Nevertheless, this study has demonstrated significant differences in ZNF880 methylation in OSCC. Therefore, testing epigenetic drugs as adjuvants in OSCC seems to be a topic worthy of exploration, which is also our next research direction.

Overall, this study conducted whole-genome DNA methylation sequencing of OSCC to obtain the methylation profile of OSCC. Based on the results of this study, we identified a large number of DMPs and DMRs. We further conducted enrichment analysis on these loci, and the results showed that the methylation of OSCC seemed to affect the life process of the tumor by participating in the PI3K-Akt signaling pathway, cancer protein polysaccharides, inflammatory mediator regulation, Rap1 signaling pathway, and so on. We then focused on exploring the impact of ZNF880 methylation levels on OSCC, demonstrating its excellent diagnostic potential and prognostic relevance. Finally, we verified the expression of ZNF880 at the gene level, confirming the association between its methylation level and gene expression.

## Conclusion

In conclusion, this study provides a comprehensive overview of the changes in genome-wide DNA methylation patterns observed in oral squamous cell carcinoma (OSCC). It was found that 37.4% of differentially methylated positions (DMPs) exhibited hypermethylation. Subsequent enrichment analysis of DMPs and differentially methylated regions (DMRs) revealed that DNA methylation in OSCC may be involved in processes such as the PI3K-Akt signaling pathway and oncoprotein polysaccharides, thereby affecting tumor growth and development. Furthermore, we hypothesize that the ZNF880 gene may serve as a novel diagnostic marker for OSCC, and its abnormal methylation may play a catalytic role in the pathogenesis of OSCC.

## Supplementary Information

Below is the link to the electronic supplementary material.

Supplementary Material 1.

Supplementary Material 2.

Supplementary Material 3.

Supplementary Material 4.

Supplementary Material 5.

## Data Availability

All data generated or analyzed during this study are included in this article. Raw data from this study is available from the corresponding author upon reasonable request.

## Abbreviations

OSCC: Oral squamous cell carcinoma
DMPs: Differential methylation probes
BMIQ: Beta-Mixture Quantile
QC: Quality control
SNPs: Single nucleotide polymorphisms
DMRs: Differential methylation regions
TSS: Transcription start site
GO: Gene ontology
KEGG: Kyoto encyclopedia of genes and genomes
BP: Biological processes
CC: Cell component
MF: Molecular function
OTSCC: Oral tongue squamous cell carcinoma
OS: Overall survival
DFS: Disease-free survival
PFS: Progression-free survival
qRT-PCR: Quantitative real-time polymerase chain reaction
