# Supplementary material for: DNA methylation analysis of the epigenome in oral squamous cell carcinoma
Source: Hum Genomics. 2026 Jan 5;20:30. doi: 10.1186/s40246-025-00899-3 (PMC12870976; doi:10.1186/s40246-025-00899-3)
Supplement: Supplementary file 1 — Supplementary Material 1. [file 40246_2025_899_MOESM1_ESM.docx]

Primers used for qRT-PCR.

| Primers for PCR | Sequence, 5'– 3' |
| --- | --- |
| ZNF880-F | AGGTATTTAAGGGTATTTTTGGTGG |
| ZNF880-R | CCTATAATCCCAACATTTTAAAAAACC |

F, forward; R, reverse.
