# Supplementary material for: DNA methylation analysis of the epigenome in oral squamous cell carcinoma
Source: Hum Genomics. 2026 Jan 5;20:30. doi: 10.1186/s40246-025-00899-3 (PMC12870976; doi:10.1186/s40246-025-00899-3)
Supplement: Supplementary file 5 — Supplementary Material 5. [file 40246_2025_899_MOESM5_ESM.docx]

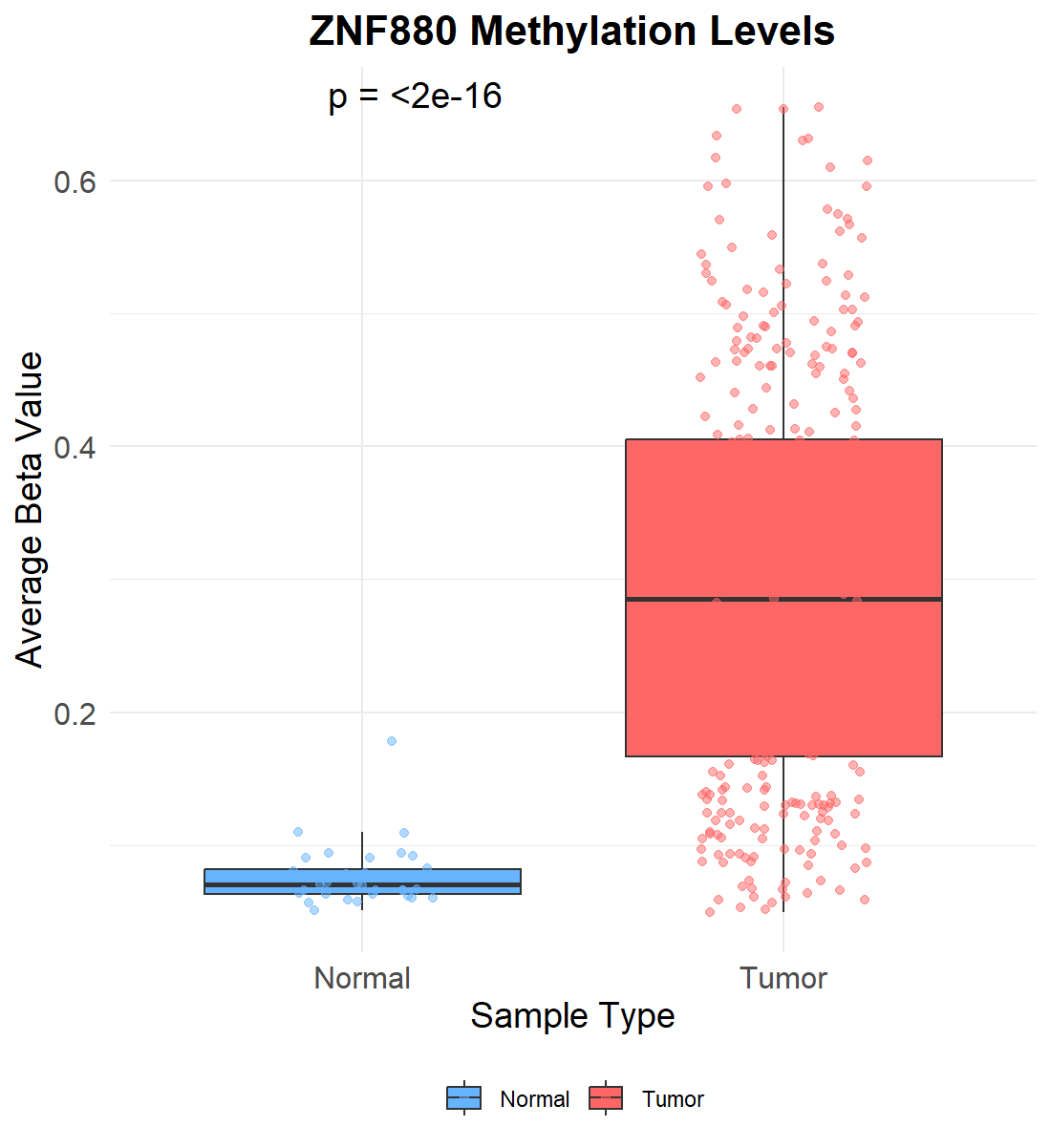


**Supplementary Figure 1: Average methylation of ZNF880 in the TCGA-OSCC cohort**

**
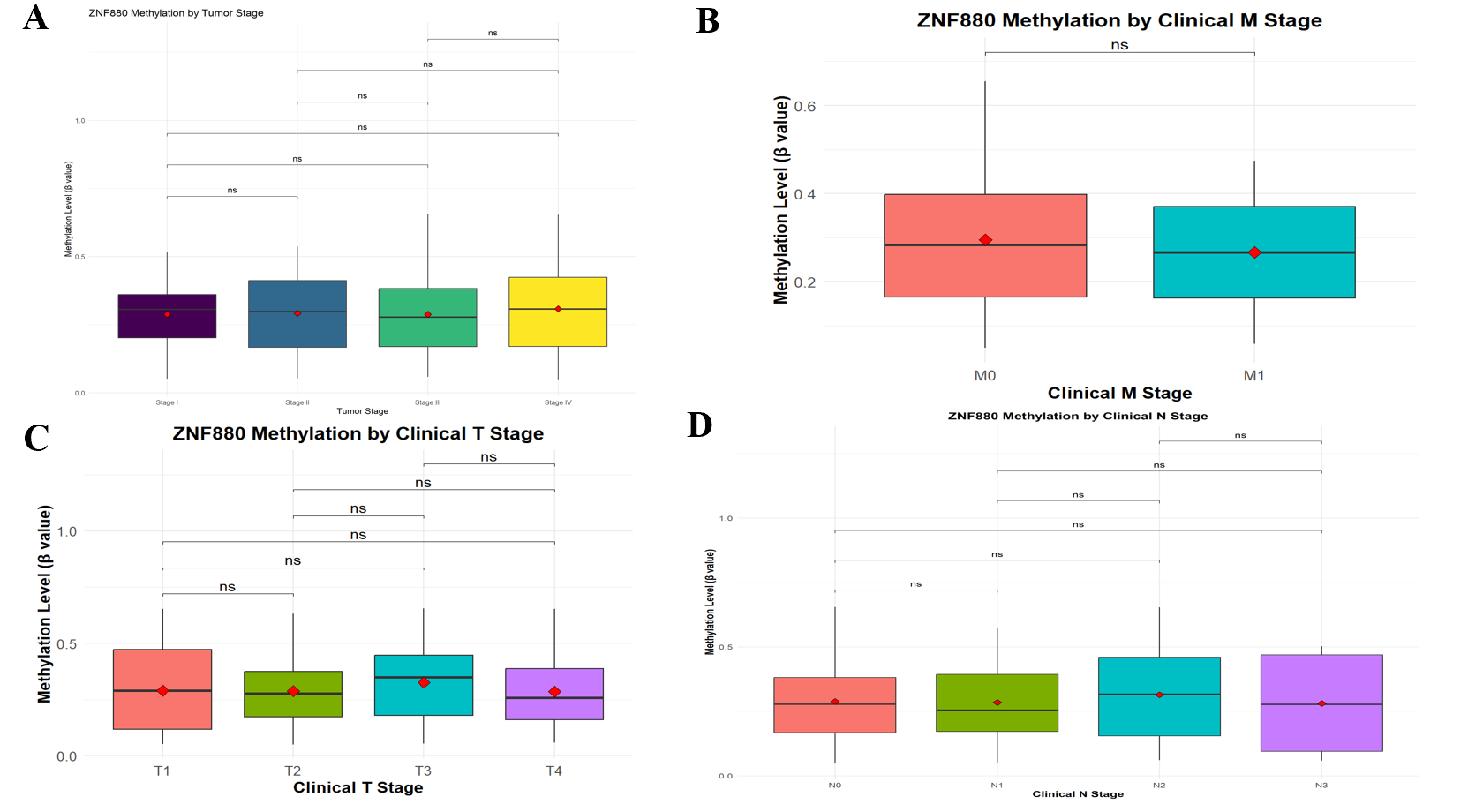
**

**Supplementary Figure 2: Correlation between the average methylation of ZNF880 and different stages in the TCGA-OSCC cohort. (A) Correlation with tumor stage, (B) Correlation with M stage, (C) Correlation with T stage, (D) Correlation with N stage**


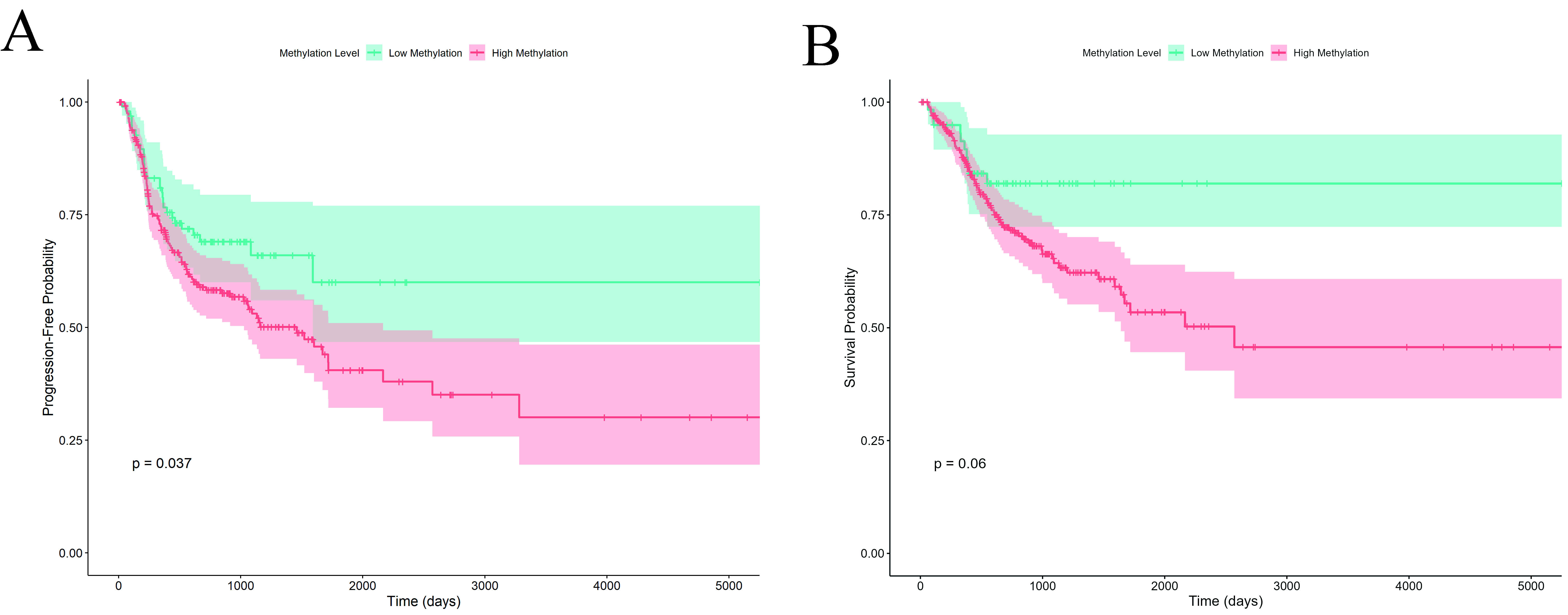


**Supplementary Figure 3: Kaplan–Meier analysis of ZNF880 methylation in the OSCC cohort from TCGA.(A) Progression-free survival (PFS) Kaplan-Meier survival curve (B)Disease-specific survival (DSS) Kaplan-Meier survival curve**


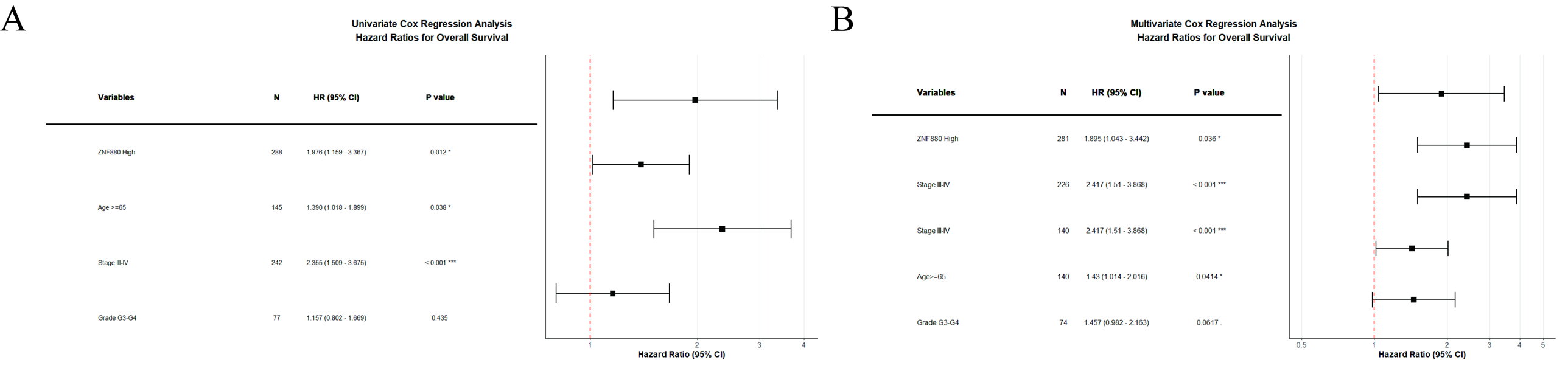


**Supplementary Figure 5: Cox regression analysis. Univariate and multivariate Cox regression (A and B) based on TCGA clinical data.****P* < .001, ***P* < .01 and **P* < .05.**
